# Long-term Risk of Hypertension After Surgical Repair of Congenital Heart Disease in Children

**DOI:** 10.1001/jamanetworkopen.2021.5237

**Published:** 2021-04-08

**Authors:** Jason H. Greenberg, Eric McArthur, Heather Thiessen-Philbrook, Michael Zappitelli, Ron Wald, Sunjay Kaushal, Derek K. Ng, Allen D. Everett, Rahul Chanchlani, Amit X. Garg, Chirag R. Parikh

**Affiliations:** 1 Division of Nephrology, Department of Pediatrics, Yale University School of Medicine, New Haven, Connecticut; 2 Clinical and Translational Research Accelerator, Department of Medicine, Yale University School of Medicine, New Haven, Connecticut; 3 ICES, London, Ontario, Canada; 4 Division of Nephrology, Department of Medicine, Johns Hopkins University, Baltimore, Maryland; 5 Division of Pediatric Nephrology, Department of Pediatrics, The Hospital for Sick Children, Toronto, Ontario, Canada; 6 Division of Nephrology, St Michael’s Hospital, University of Toronto, Toronto, Ontario, Canada; 7 Department of Surgery, University of Maryland, Baltimore; 8 Department of Epidemiology, Johns Hopkins University, Baltimore, Maryland; 9 Department of Pediatrics, Johns Hopkins University, Baltimore, Maryland; 10 Division of Nephrology, Department of Pediatrics, McMaster Children's Hospital, McMaster University, Hamilton, Ontario, Canada; 11 Department of Medicine, Western University, London, Ontario, Canada

## Abstract

**Importance:**

The long-term risk of hypertension in children after surgery for congenital heart disease (CHD) is unclear.

**Objective:**

To assess the incidence of hypertension after cardiac surgery in children with CHD.

**Design, Setting, and Participants:**

A multicenter retrospective matched cohort study was conducted in Ontario, Canada, using administrative databases. A total of 3600 children with surgical repair of CHD were matched to 10 children (n = 36 000) from the general population without CHD on age, sex, index date, rurality, and neighborhood income.

**Main Outcomes and Measures:**

Diagnosis of hypertension over a median follow-up time of 9.8 years (interquartile range, 6.8-12.9 years) after surgery. The last follow-up was March 31, 2019.

**Results:**

Overall, in 3600 children with surgical repair of CHD, the median age at first surgery was 150 days (interquartile range, 40-252 days) and 2005 (55.7%) were boys. During follow-up, 445 (12.4%) children with surgical repair of CHD developed hypertension compared with 398 (1.1%) in the matched control group. The incidence rate of hypertension in children who received surgery for CHD was 141.3 (95% CI, 128.8-155.1) per 10 000 person-years compared with children in the matched control group, who had a rate of 11.1 (95% CI, 10.1-12.3) per 10 000 person-years. The risk of hypertension was higher in children with index surgical dates at an age of less 150 days compared with those who had surgical dates at an age of 150 days or older (*P* = .006 for interaction). The risk of hypertension was increased in children with more complex surgery, particularly children with hypoplastic left heart syndrome (49 of 140 [35.0%]), and in children who received dialysis (22 of 126 [17.5%]; hazard ratio, 1.67; 95% CI, 1.09-2.56) during the index cardiac surgery hospitalization.

**Conclusions and Relevance:**

The incidence of long-term hypertension in this study was 12 times higher in children with surgical repair of CHD compared with children in the matched control group. The findings suggest that interventions aimed at reducing the long-term risk of hypertension after cardiac surgery in this population are needed.

## Key Points

**Question:**

What is the incidence of hypertension after cardiac surgery in children with congenital heart disease?

**Findings:**

In this cohort study of 3600 children with surgical repair of congenital heart disease who were matched to 36 000 children from the general population without congenital heart disease, the incidence rate of hypertension in children who underwent cardiac surgery was 12 times higher than the incidence in matched children in the control group. The risk of hypertension was particularly increased in children with hypoplastic left heart syndrome and in those who received dialysis during the index cardiac surgery hospitalization.

**Meaning:**

The findings of this study suggest that interventions aimed at reducing the long-term risk of hypertension in children with congenital heart disease following cardiac surgery are needed.

## Teaser

This cohort study examines the risk of hypertension over time in children who have received surgery for congenital heart disease.

## Introduction

Congenital heart disease (CHD) is the most common type of birth defect, affecting approximately 2.4 million individuals in the US.^1,2^ Although many children with CHD require no surgical intervention, approximately 1 in 4 children with CHD require cardiac surgery.^1^ Advances in the diagnosis and treatment of CHD have led to improved survival rates and quality of life for children after surgical repair of the cardiac defect. Because of this improved survival, there is now an intensified focus on assessment and prevention of long-term cardiovascular disease and hypertension.

Previous research has documented the pathologic changes to the cardiovascular system and kidneys after cardiac surgery.^3^ Cardiopulmonary bypass can lead to injury and remodeling of the cardiovascular system and kidneys as well as subsequent complications, such as acute kidney injury, chronic kidney disease, and hypertension.^4,5,6,7,8^ In a multicenter, prospective cohort study of 131 children who underwent cardiac surgery for repair of CHD, hypertension was 10 times more prevalent at 5 years of follow-up than in the general pediatric population.^9^ The adverse cardiovascular outcomes associated with hypertension are especially concerning in children with CHD, who are already at higher risk of arrhythmia and heart failure.^10^

Studying the cumulative incidence of hypertension after cardiac surgery is important to identify risk factors that could be modified by early intervention and treatment. Also, to our knowledge, there are no current consensus recommendations to guide the monitoring and treatment of hypertension after cardiac surgery for CHD. In the present study, we examined the risk of hypertension after surgical repair of CHD in a large Canadian province. We hypothesized that surgical repair of CHD would be associated with an increased risk of hypertension. Moreover, because this database contains individuals with multiple types of CHD, we estimated that the risk of hypertension would be more common in children with more severe types of congenital heart defects.

## Methods

### Study Design

We conducted a retrospective matched cohort study. Our study used administrative health care databases linked using unique, encoded identifiers and analyzed at ICES, a nonprofit organization in Ontario, Canada, that uses data collected through routine administration of Ontario’s public health care system for health services research. The 14.6 million residents of Ontario, Canada, have universal access to medical care. The risk of mortality and end-stage kidney disease in this cohort has been previously studied, although the outcomes were examined only until March 15, 2015.^11^ This study followed the Strengthening the Reporting of Observational Studies in Epidemiology (STROBE) reporting guideline. Our study of Ontario administrative health care databases did not require research ethics board approval or informed consent per section 45 of Ontario’s Personal Health Information Protection Act.

### Data Sources

For this study, data from 7 linked databases were examined. The MOMBABY database contains inpatient admission records for mothers and their newborn children in Ontario and links mothers and their newborns according to the mother and newborn medical record numbers. The Registered Persons Database has demographic information and statistics on all people with a valid health card residing in Ontario. Hospitalization and the associated procedural records, baseline characteristics, and outcomes were obtained from the Canadian Institute for Health Information Discharge Abstract Database. Same-day surgeries and their associated baseline characteristics and outcomes were obtained from the Canadian Institute for Health Information same-day surgery database. The ICES-derived physician database detailed the medical professional characteristics. The Ontario Health Insurance Plan database captures physician claims data, used in this study for exclusions, baselines, and outcomes. The Canadian Organ Replacement Register is a nationwide Canadian database that was used to identify patients with end-stage kidney disease.^12,13^

Congenital heart disease–related surgeries were identified using Ontario Health Insurance Plan surgical billing codes. The Canadian Classification of Health Interventions was used to capture procedures (eTable 1 in the Supplement) and the *International Statistical Classification of Diseases, Tenth Revision* (*ICD-10*) was used to identify the type of CHD. Data fields with 5 or fewer patients were suppressed (reported as ≤5).

### Population

Individuals who were born in Ontario between April 1, 2002, and March 31, 2015, were identified.^11^ We identified CHD as any CHD-related surgery within 10 years of birth. The study entry date was considered the surgery date that is also referred to as the index date for each patient. In addition, individuals had to have a CHD diagnosis before or after their surgery date. We excluded neonates and children with a patent ductus arteriosus ligation and no other surgical code during their index hospitalization. We also excluded neonates and children with hypertension or end-stage kidney disease before the index date. For those with more than 1 eligible CHD-related surgery, their first surgery was used as the index date.

We defined the severity of CHD using consensus guidelines. Severe CHD included atrioventricular septal defect, tetralogy of Fallot, univentricular heart, transposition complex, truncus arteriosus, and hypoplastic left heart syndrome.^14^ We used the Society of Thoracic Surgeons–European Association for Cardio-Thoracic Surgery (STAT) scores to classify the complexity of cardiac surgery as 1 (eg, repair of atrial septal defect and coarctation repair), 2 (eg, transposition of the great arteries repair and total repair of tetralogy of Fallot), 3 (eg, arterial switch operation and the Fontan procedure), 4 (eg, mitral valve replacement and aortic arch repair), and 5 (eg, Norwood procedure).^15^ To provide a comparison for the risk of hypertension in the general pediatric population, we assembled a cohort of children without CHD. We considered newborns with no CHD diagnosis as potential unexposed control participants. For the unexposed control participants, a random index date was assigned based on the distribution of times from birth to the index date of surgery in the group that received surgery for CHD. We matched each patient with surgery for CHD with 10 children in the control group using a greedy algorithm without replacement on index date (±365 days), age at index date (±90 days), sex, neighborhood income quintile, and rural residence (municipality with a population <10 000).

### Study Outcomes

Children were followed up until death, hypertension, or the end of data availability (March 31, 2019). The primary outcome was time to first diagnosis of hypertension. Hypertension was defined as the first evidence of an inpatient or outpatient hypertension *ICD-10* diagnostic code during follow-up after the index hospitalization. The *ICD-10* diagnostic codes are detailed in eTable 2 in the Supplement, along with information on validation of the codes. In adults, the specificity of the diagnostic codes was 92%, the sensitivity was 72%, and the positive predictive value was 87%. Loss to follow-up was minimal, as annual emigration from Ontario is estimated at 0.1%.^16^

### Statistical Analysis

Continuous variables are reported as mean (SD) or median (interquartile range [IQR]) depending on the distribution of the data. Categorical variables are reported as numbers and percentages. We compared baseline characteristics between the group that received surgery for CHD and children in the matched control group using standardized differences, with a standardized difference greater than 10% considered meaningful. All variables in the analysis were complete except for neighborhood income quintile and rural residence, which were missing for less than 0.5% of the cohort.

We used Cox proportional hazards regression models, stratified on matched sets, to obtain hazard ratios (HRs), and we tested the proportionality assumption by adding a time-dependent exposure covariate to the model. Death was treated as a censoring event because the proportion was low (3.9% among those with CHD and 0.1% for the comparison cohort). Because preterm birth (<37 weeks) was more common among those with CHD, we estimated the association between cardiac surgery and hypertension after adjusting for preterm birth in the Cox proportional hazards regression model. Low birth weight had high colinearity with preterm birth (*r* > 0.6) and was thus not included in multivariable models. We also performed subgroup analyses to examine the outcomes of individuals stratified by sex, specific CHD diagnoses, younger than 150 days, and neonate status (age <28 days). To assess the potential contribution of receipt of dialysis for acute kidney injury during the index hospitalization to long-term hypertension, we modeled receipt of dialysis during the index hospitalization as a covariate in a Cox proportional hazards regression model restricted to those with CHD. We performed an additional analysis to assess the association of incident hypertension (as the time-varying independent variable) and time to end-stage kidney disease (as the outcome) among those with CHD only and time from surgery as the time scale. We also performed a sensitivity analysis looking at the cumulative incidence of hypertension when the diagnosis of hypertension was made at least 1 year after the index date. We also studied the association of multiple surgeries in the first year with hypertension. Follow-up time for this analysis began on day 366, and we restricted the analysis to those who survived the first year after discharge from their index hospitalization to avoid immortal time bias. The Kaplan-Meier product-limit estimator was used to nonparametrically derive survival functions. A 2-sided *P* value <.05 was considered statistically significant, and analyses were performed using SAS, version 9.4 (SAS Institute Inc).

## Results

We matched 3600 children who received surgical repair for CHD to 36 000 matched children without CHD (eFigure in the Supplement). The baseline characteristics for the cohort are presented in Table 1. The median age at the index cardiac surgery hospitalization was 150 days (IQR, 40-252 days), 2005 children (55.7%) were boys, and 1595 (44.3%) were girls. Children who received surgical repair for CHD had a lower gestational age, lower birth weight, more chromosomal abnormalities, more noncardiac malformations, and more malformations of the urinary system compared with controls. According to administrative *ICD-10* diagnosis codes, 1866 children (52%) were classified as having severe CHD. To classify the complexity of surgery received by the children with CHD, the STAT mortality score was used (Table 1).

**Table 1.** Characteristics of Patients in the Study

Variable	Congenital heart disease, No. (%)	
	Yes (n=3600)	No (n=36 000
Demographic characteristic		
Age, median (IQR), y	0.4 (0.1-0.7)	0.4 (0.1-0.7)
Sex		
Male	2005 (56)	20 050 (56)
Female	1595 (44)	15 950 (44)
Rural residence	386 (10.7)	3860 (10.7)
Income quintile		
1 (lowest)	790 (21.9)	7900 (21.9)
2	721 (20.0)	7210 (20.0)
3	751 (20.9)	7510 (20.9)
4	765 (21.3)	7650 (21.3)
5 (highest)	573 (15.9)	5730 (15.9)
Year of surgery (reference date for normal)		
2002-2004	566 (15.7)	5652 (15.7)
2005-2007	781 (21.7)	7827 (21.7)
2008-2010	958 (26.6)	9579 (26.7)
2011-2013	931 (25.9)	9310 (25.9)
2014-2015	364 (10.1)	3632 (10.1)
Maternal age, median (IQR), y	32 (28-36)	31 (27-35)
Gestational age, wk		
Mean (SD)	38 (2.42)	38.89 (2.07)
Median (IQR)	38 (37-40)	39 (38-40)
<37, Preterm	652 (18.1)	2694 (7.5)
<32, Very preterm	98 (2.7)	263 (0.7)
<28, Extremely preterm	29 (0.8)	67 (0.2)
Birth weight, g		
Mean (SD)	3066 (711)	3377.3 (556.33)
Median (IQR)	3140 (2670-3532)	3400 (3053-3730)
<2500, Low	681 (18.9)	2042 (5.7)
<1500, Very low	104 (2.9)	171 (0.5)
Multiple birth		
Yes	190 (5.3)	1074 (3.0)
Chromosomal anomaly	470 (13.1)	22 (0.1)
Artificial insemination	35 (1.0)	229 (0.6)
Chronic kidney disease	10 (0.3)	16 (<0.1)
Surgery hospitalization characteristics		
Any congenital heart disease diagnosis	3600 (100)	NA
Severe congenital heart disease diagnosis	1866 (51.8)	NA
STAT score^a^		
1	1072 (29.8)	NA
2	1183 (32.9)	NA
3	398 (11.1)	NA
4	749 (20.8)	NA
5	198 (5.4)	NA
Hospital length of stay, d		
Mean (SD)	24.66 (66.83)	NA
Median (IQR)	10 (5-23)	NA
Time from surgery to discharge, d		
Mean (SD)	19.71 (63.26)	NA
Median (IQR)	8 (5-17)	NA
ICU length of stay, d		
Mean (SD)	11.68 (25.00)	NA
Median (IQR)	5 (2-11)	NA
Time on ventilation, d		
Mean (SD)	9.31 (20.73)	NA
Median (IQR)	4 (2-9)	NA
Dialysis during index hospitalization	126 (3.5)	NA

Abbreviations: ICU, intensive care unit; IQR, interquartile range; NA, not applicable; STAT, Society of Thoracic Surgeons–European Association for Cardio-Thoracic Surgery.

^a^ The STAT score is used to categorize the complexity of cardiac surgery. STAT score 1 includes surgeries such as repair of atrial septal defect and coarctation repair; score 2 includes surgeries such as transposition of the great arteries repair and total repair of tetralogy of Fallot; score 3 includes surgeries such as an arterial switch operation and the Fontan procedure; score 4 includes a mitral valve replacement and an aortic arch repair; and score 5 includes surgeries such as a Norwood procedure.

The most common types of CHD were ventricular septal defect (575 [16%]), tetralogy of Fallot (449 [12%]), atrioventricular septal defect (426 [12%]), and coarctation of the aorta (377 [10%]) (eTable 3 in the Supplement). The most common procedures during the surgical hospitalization were closure of an atrial septal defect (1191 [33%]) and closure of a ventricular septal defect (1109 [31%]). The median length of stay in the intensive care unit was 5 days (IQR, 2-11 days) and median length of stay in the hospital post surgery was 8 days (IQR, 5-17 days). During the index cardiac surgery hospitalization, 126 patients (4%) received dialysis.

**Figure 1.**
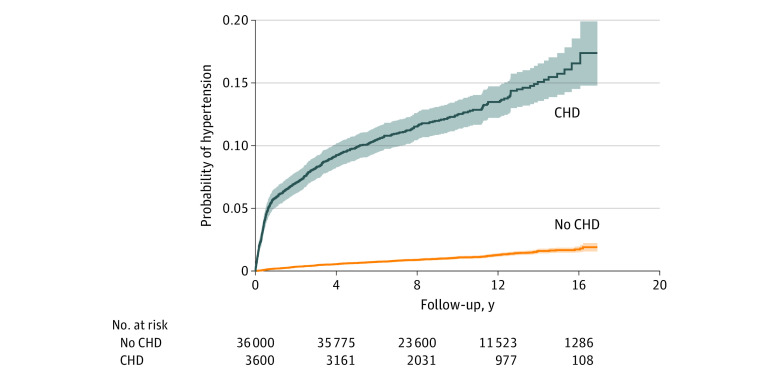
Time-to-Event Analysis of Hypertension Shaded area indicates 95% CIs. CHD indicates congenital heart disease.

During the study period, these children who received surgery for CHD contributed a total of 388 624 observed person-years. The median follow-up was 9.8 years (IQR, 6.8-12.9 years) after surgery. Compared with children in the matched control group, those who had surgery for CHD had more frequent visits with pediatricians, cardiologists, and nephrologists (eTable 4 in the Supplement).^11^ In children who received surgery for CHD, the incidence rate was 141.3 (95% CI, 128.8-155.1) per 10 000 person-years and the cumulative incidence of hypertension was 12.4%, compared with 11.1 (95% CI, 10.1-12.3) per 10 000 person-years and 1.1% in children in the matched control group (unadjusted HR, 12.5; 95% CI, 10.9-14.4) (Table 2) (Figure 1). The HR of hypertension was largely unchanged after adjustment for preterm birth status (adjusted HR, 12.7; 95% CI, 11.0-14.7).

**Table 2.** Risk of Hypertension in the Full Cohort and Subgroups

Status	No.	Events, No. (%)	Incidence rate per 10 000 person-years	HR (95% CI)	*P* value for interaction
Full cohort					
No CHD	36 000	398 (1.1)	11.1	1 [Reference]	NA
CHD	3600	445 (12.4)	141.3	12.5 (10.9-14.4)	
Age at surgery, <150 d					
No CHD	17 450	211 (1.2)	18.9	1 [Reference]	.004
CHD	1745	275 (15.8)	288.4	14.8 (12.3-17.9)	
Age at surgery, ≥150 d					
No CHD	18 550	187 (1.0)	18.1	1 [Reference]	NA
CHD	1855	170 (9.2)	175.9	10.0 (8.1-12.4)	
Not neonate at surgery					
No CHD	28 590	303 (1.1)	10.8	1 [Reference]	.50
CHD	2859	320 (11.2)	127.8	11.7 (9.9-13.7)	
Neonate at surgery					
No CHD	5000	67 (1.3)	13.1	1 [Reference]	NA
CHD	500	76 (15.2)	173.3	13.3 (9.4-18.7)	
Not preterm					
No CHD	13 770	147 (1.1)	10.8	1 [Reference]	.65
CHD	1377	168 (12.2)	140.2	13.1 (10.4-16.4)	
Preterm					
No CHD	22 230	251 (1.1)	11.4	1 [Reference]	NA
CHD	2223	277 (12.5)	142.0	12.2 (10.2-14.5)	
Female					
No CHD	15 950	168 (1.1)	10.7	1 [Reference]	.17
CHD	1595	171 (10.7)	121.6	11.1 (9.0-13.8)	
Male					
No CHD	20 050	230 (1.2)	11.5	1 [Reference]	NA
Table	2005	274 (13.7)	157.3	13.6 (11.3-16.3)	
STAT score^a^					
1					.02
No CHD	10720	101 (0.9)	9.6	1 [Reference]	
CHD	1072	71 (6.6)	70.6	7.3 (5.4-9.9)	
2					
No CHD	11830	122 (1.0)	10.5	1 [Reference]	
CHD	1183	132 (11.2)	126.0	11.9 (9.2-15.3)	
3					
No CHD	3980	49 (1.2)	12.3	1 [Reference]	
CHD	398	31 (7.8)	85.2	7.00 (4.4-11.1)	
4					
No CHD	7490	97 (1.3)	13.3	1 [Reference]	
CHD	749	156 (20.8)	272.7	19.6 (15.0-25.8)	
5					
No CHD	1980	29 (1.5)	12.8	1 [Reference]	NA
CHD	198	55 (27.8)	344.9	23.8 (14.6-38.7)	

Abbreviations: CHD, congenital heart disease; HR, hazard ratio; NA, not applicable; STAT, Society of Thoracic Surgeons–European Association for Cardio-Thoracic Surgery.

**Figure 2.**
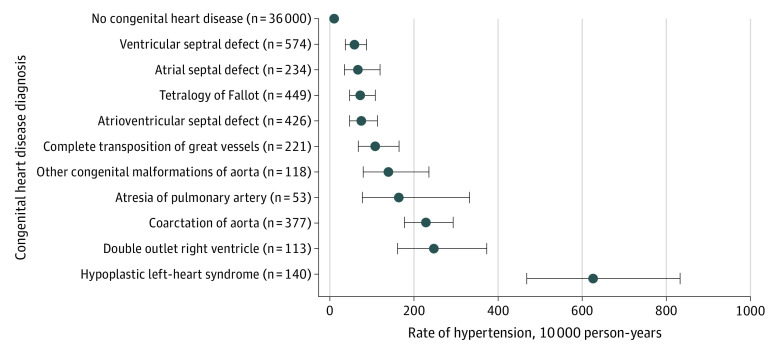
Long-term Risk of Hypertension After Cardiac Surgery by Type of Congenital Heart Disease Error bars indicate 95% CIs.

**Figure 3.**
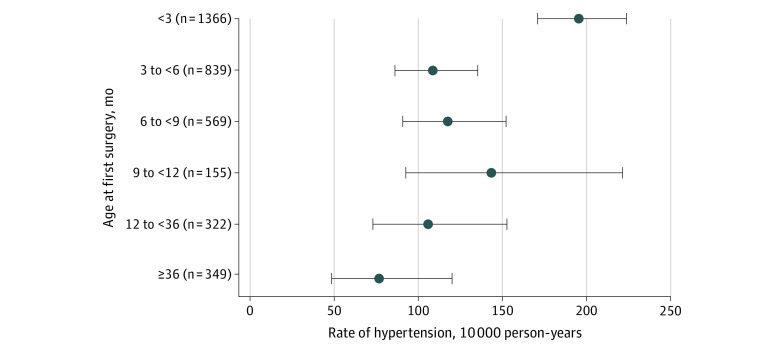
Long-term Risk of Hypertension After Cardiac Surgery by Age at the Time of Surgery Error bars indicate 95% CIs.

During follow-up, 35.0% (49 of 140) of the children with hypoplastic left heart syndrome developed hypertension. Other types of CHD with a high risk of hypertension included double-outlet right ventricle (21.2% [24 of 113]), coarctation of the aorta (19.1% [72 of 377]), and atresia of the pulmonary artery (15.1% [8 of 53]) (Figure 2) (eTable 5 in the Supplement). Children with index surgical dates younger than 150 days had an increased risk of hypertension compared with individuals with surgical dates at age 150 days or older (*P* = .006 for interaction) (Table 2). The higher risk of hypertension in this group was primarily associated with the increased incidence rate in those who underwent surgery when they were younger than 3 months, with an incidence rate in this age group of 195.4 (95% CI, 171.8-222.3) per 10 000 person-years (Figure 3).

In addition, children with a STAT mortality score of 4 (HR, 19.64; 95% CI, 14.98-25.75) or 5 (HR, 23.79; 95% CI, 14.62-38.70) had an increased risk of hypertension compared with those with the other STAT scores (Table 2). There was no significant interaction by sex, neonate vs nonneonate at the time of surgery, or children born preterm vs full term for the primary outcome of hypertension (Table 2). Children who received at least 1 additional cardiac surgery during the first year of follow-up were at a 3-fold higher risk of hypertension (HR, 3.1; 95% CI, 2.2-4.3) compared with those who received only 1 cardiac surgery. In addition, children who had surgical repair of CHD and received dialysis (17.5% [22 of 126]) during their index cardiac surgery hospitalization experienced a higher risk of hypertension (HR, 1.67; 95% CI, 1.09-2.56) compared with those who did not receive dialysis (12.2% [423 of 3474]).

During follow-up, 79 (2.2%) children with CHD and hypertension developed end-stage kidney disease. Compared with children who did not develop hypertension, those who developed hypertension were at an increased risk of end-stage kidney disease (HR, 1.90; 95% CI, 1.03-3.53). Furthermore, we found that when our analysis was restricted to diagnoses of hypertension made at least a year after the index date, the HR was lower at 8.2 (95% CI, 6.9-9.8) for children who received surgery for CHD vs children in the matched control group compared with diagnoses of hypertension made at any time after the index date (HR, 12.5; 95% CI, 10.9-14.4).

## Discussion

We observed that children with a surgical repair of CHD in this study had a 12-fold higher hazard of hypertension compared with children in the matched control group. The cumulative risk of hypertension appeared to increase at the fastest rate during the first 2 years after surgery to 5% of children with CHD. The risk of hypertension then gradually increased over the full follow-up time. In stratified analyses, we observed that the youngest children (ie, age <150 days at the time of surgery) were at the greatest risk of hypertension. For individual CHD diagnoses, the highest risk of hypertension was in children with hypoplastic left heart syndrome. However, several other diagnoses, including double-outlet right ventricle, coarctation of the aorta, and atresia of the pulmonary artery, were also associated with high rates of hypertension compared with children in the matched control group.

To our knowledge, this is the first study with long-term follow-up to describe the risk of hypertension in a large cohort of children who underwent cardiac surgery. The increased incidence of hypertension is especially significant because hypertension may be a harbinger of adverse cardiovascular outcomes later in adulthood, as seen in earlier research.^17^ Bauer et al^18^ found that 21% (113 of 539) of adults with CHD had hypertension. Martínez-Quintana et al^19^ reported that 12% (101 of 818) of adults with CHD had hypertension compared with 10% (186 of 1955) of the general population. The 12% prevalence in adults with CHD reported by Martínez-Quintana et al^19^ is similar to the 12.6% reported in our study, although only 1.1% of the children in our control group had hypertension. Our research suggests that the excess burden of hypertension recognized in adults with CHD may start early during childhood, allowing an earlier time for intervention.^3,4^

Hypertension may have several underlying mechanisms, including a disturbance of the cardiac receptors during surgery leading to sympathetic activation and postoperative development of clinical or subclinical kidney injury.^20,21,22^ In addition, an upregulated renin-angiotensin system and elevated atrial natriuretic peptide, B-type natriuretic peptide, and norepinephrine levels may persist for years after surgical repair of CHD.^8,23^ Even patients with a surgically closed atrial septal defect were found to have persistently elevated B-type natriuretic peptide levels decades after repair.^24,25^ These neurohormonal derangements represent an attempt to preserve hemodynamic status and renal perfusion; however, these derangements may substantially affect blood pressure, intraglomerular hemodynamics, and tubular function.^3^ Neurohormonal activation and its influences on cardiovascular and kidney function may partially explain the varying rates of hypertension in different types of CHD. Patients with coarctation of the aorta who have residual morphologic obstruction after surgery are at increased risk of developing hypertension.^26^ In addition, maternal hypertension in pregnancy is associated with an increased risk of CHD in offspring, which may suggest that genetic factors provide a link between hypertension and CHD.^23^ Previous research on children with CHD in Ontario documented that the risk of end-stage kidney disease was high compared with the general population, with the highest risk in children with hypoplastic left heart syndrome.^11^ Similarly, in the present study, the highest risk of hypertension was observed in children with hypoplastic left heart syndrome. We also observed that children who received dialysis during their index cardiac surgery hospitalization were at an increased risk for developing hypertension.

In our study, infants who underwent their index surgery at younger than 150 days had a 65% higher incidence rate of hypertension than those aged 150 days or older. Moreover, we observed that this high risk of hypertension in the youngest children was associated with those who have surgery at younger than 3 months. Our findings are consistent with those reported by Huynh et al,^27^ who found that the long-term risk of hypertension after repair of congenital heart defects in neonates was 30%, compared with 0.8% in healthy Canadian children. Age at surgery may be a surrogate for the severity of the congenital heart defect because infants who urgently require surgical repair for severe disease cannot delay surgery to an older age. In addition, hypertension is common in specific types of CHD that are more likely to be repaired at an early age, such as coarctation of the aorta and hypoplastic left heart syndrome. Also, the immature kidney function and limited physiologic reserve of neonates may contribute to their long-term risk of hypertension after cardiac surgery.^28^ However, the cumulative risk of hypertension is high even among those who underwent their index surgery at age 150 days or older. In addition, in children with surgical repair of CHD, we observed an association between incident hypertension and an increased risk of end-stage kidney disease.

### Limitations

Our study has limitations. We did not have access to individual blood pressure measurements or the prescription of antihypertensive medications and therefore relied on administrative diagnostic codes to identify patients with hypertension. Physicians use clinical judgment when applying accepted diagnostic criteria for hypertension, and administratively coded diagnoses may have variable clinical significance. Another limitation is that patients with CHD regularly interacted with medical professionals who may measure blood pressure and document elevated blood pressure as a part of the visit. It is possible that these more frequent measures of blood pressure in children with CHD may have led to an ascertainment bias due to more health care encounters in the cardiac surgery group. Therefore, hypertension was more likely to be detected in patients with CHD than in the matched control group. It is also conceivable that hypertension is underdiagnosed in the unexposed controls because their blood pressure is only monitored at routine health care visits as infrequently as once per year. Moreover, blood pressure is not monitored in most healthy children before the age of 3 years, whereas it is routinely checked at all ages in children after cardiac surgery. The observed risk of hypertension for unexposed controls in our study was 1.1%, which is lower than the estimated prevalence of hypertension in the general pediatric population of 3.5%.^17,29^ Our analysis was conducted using administrative diagnosis codes, and thus we were unable to perform in-depth examinations of heterogeneous conditions with distinct physiologic characteristics, such as double-outlet right ventricle. We also acknowledge the multiple statistical comparisons performed in our investigation and the risk of chance (false-positive) findings. The strengths of our study include that multiple centers across Ontario were represented and that there was minimal loss to follow-up.^20^

## Conclusions

We observed a high incidence of hypertension in children with CHD after cardiac surgery. This risk of hypertension appeared to be higher for those who had their first cardiac surgery before age 150 days. We identified that this increased burden of hypertension appears to affect all types of congenital heart defects but may be greatest in those with hypoplastic left heart syndrome. Our results have implications for the many children with CHD who undergo cardiac surgery and have routine follow-up. Early detection and treatment of hypertension are needed in this vulnerable population. Our findings provide data to possibly enhance recommendations for blood pressure monitoring and follow-up of children after cardiac surgery. To better characterize the burden of hypertension in children with prior surgery for CHD, future research should use rigorous casual (ie, single) measurements in the clinic and ambulatory blood pressure monitoring as well as neurohormonal assessments to better understand the potential mechanisms of hypertension. These future studies should be conducted in large multicenter cohorts to better characterize hypertension risk in children with specific types of CHD. In addition, clinical trials are needed to test interventions to reduce the risk of incident hypertension and determine optimal blood pressure targets.

## Notes

Supplement. **eTable 1.** Cohort Build and Baseline Characteristic Codes **eTable 2.** Outcome Codes **eTable 3.** *ICD-10* CHD Diagnosis and Surgical Billing Codes **eTable 4.** Health Care Utilization Among Those With Congenital Heart Disease Receiving Surgery vs Matched Controls **eTable 5.** Long-Term Risk of Hypertension by Type of Congenital Heart Disease **eFigure.** Patient Selection

## References

[1] Hoffman JI, Kaplan S. The incidence of congenital heart disease. J Am Coll Cardiol. 2002;39(12):1890-1900. doi:10.1016/S0735-1097(02)01886-7 12084585

[2] Gilboa SM, Devine OJ, Kucik JE, . Congenital heart defects in the United States: estimating the magnitude of the affected population in 2010. Circulation. 2016;134(2):101-109. doi:10.1161/CIRCULATIONAHA.115.019307 27382105 PMC4942347

[3] Morgan C, Al-Aklabi M, Garcia Guerra G. Chronic kidney disease in congenital heart disease patients: a narrative review of evidence. Can J Kidney Health Dis. 2015;2:27. doi:10.1186/s40697-015-0063-8 26266042 PMC4531493

[4] Magri P, Rao MA, Cangianiello S, . Early impairment of renal hemodynamic reserve in patients with asymptomatic heart failure is restored by angiotensin II antagonism. Circulation. 1998;98(25):2849-2854. doi:10.1161/01.CIR.98.25.2849 9860786

[5] Ishikawa S, Miyauchi T, Sakai S, . Elevated levels of plasma endothelin-1 in young patients with pulmonary hypertension caused by congenital heart disease are decreased after successful surgical repair. J Thorac Cardiovasc Surg. 1995;110(1):271-273. doi:10.1016/S0022-5223(05)80036-4 7609554

[6] Lang RE, Unger T, Ganten D, Weil J, Bidlingmaier F, Dohlemann D. Alpha atrial natriuretic peptide concentrations in plasma of children with congenital heart and pulmonary diseases. BMJ (Clin Res Ed). 1985;291(6504):1241. doi:10.1136/bmj.291.6504.1241 2933120 PMC1417054

[7] Schrier RW, Abraham WT. Hormones and hemodynamics in heart failure. N Engl J Med. 1999;341(8):577-585. doi:10.1056/NEJM199908193410806 10451464

[8] Greenberg JH, Coca S, Parikh CR. Long-term risk of chronic kidney disease and mortality in children after acute kidney injury: a systematic review. BMC Nephrol. 2014;15:184. doi:10.1186/1471-2369-15-184 25416588 PMC4251927

[9] Greenberg JH, Zappitelli M, Devarajan P, ; TRIBE-AKI Consortium. Kidney outcomes 5 years after pediatric cardiac surgery: the TRIBE-AKI study. JAMA Pediatr. 2016;170(11):1071-1078. doi:10.1001/jamapediatrics.2016.1532 27618162 PMC5476457

[10] Billett J, Cowie MR, Gatzoulis MA, Vonder Muhll IF, Majeed A. Comorbidity, healthcare utilisation and process of care measures in patients with congenital heart disease in the UK: cross-sectional, population-based study with case-control analysis. Heart. 2008;94(9):1194-1199. doi:10.1136/hrt.2007.122671 17646191

[11] Parikh CR, Greenberg JH, McArthur E, . Incidence of ESKD and mortality among children with congenital heart disease after cardiac surgery. Clin J Am Soc Nephrol. 2019;14(10):1450-1457. doi:10.2215/CJN.00690119 31501090 PMC6777584

[12] Agha MM, Glazier RH, Moineddin R, Moore AM, Guttmann A. Socioeconomic status and prevalence of congenital heart defects: does universal access to health care system eliminate the gap? Birth Defects Res A Clin Mol Teratol. 2011;91(12):1011-1018. doi:10.1002/bdra.22857 22002854

[13] Garg AX, McArthur E, Lentine KL; Donor Nephrectomy Outcomes Research (DONOR) Network. Gestational hypertension and preeclampsia in living kidney donors. N Engl J Med. 2015;372(15):1469-1470. 25853753 10.1056/NEJMc1501450

[14] Marelli AJ, Mackie AS, Ionescu-Ittu R, Rahme E, Pilote L. Congenital heart disease in the general population: changing prevalence and age distribution. Circulation. 2007;115(2):163-172. doi:10.1161/CIRCULATIONAHA.106.627224 17210844

[15] Cavalcanti PE, Sá MP, Santos CA, . Stratification of complexity in congenital heart surgery: comparative study of the Risk Adjustment for Congenital Heart Surgery (RACHS-1) method, Aristotle basic score and Society of Thoracic Surgeons–European Association for Cardio-Thoracic Surgery (STS-EACTS) mortality score. Rev Bras Cir Cardiovasc. 2015;30(2):148-158. doi:10.5935/1678-9741.20150001 26107445 PMC4462959

[16] Ontario Ministry of Finance. Ontario Population Projections Update: Spring 2017, Based on the 2011 Census. Ontario Ministry of Finance; 2017.

[17] Theodore RF, Broadbent J, Nagin D, . Childhood to early-midlife systolic blood pressure trajectories: early-life predictors, effect modifiers, and adult cardiovascular outcomes. Hypertension. 2015;66(6):1108-1115. doi:10.1161/HYPERTENSIONAHA.115.05831 26558818 PMC4646716

[18] Bauer UMM, Körten MA, Diller GP, . Cardiovascular risk factors in adults with congenital heart defects—recognised but not treated? an analysis of the German National Register for Congenital Heart Defects. Int J Cardiol. 2019;277:79-84. doi:10.1016/j.ijcard.2018.08.009 30100225

[19] Martínez-Quintana E, Rodríguez-Hernández JL, Rodríguez-González F, . Cardiovascular risk factors and arterial thrombotic events in congenital heart disease patients. Int J Clin Pract. 2019;73(9):1-8. doi:10.1111/ijcp.13378 31141298

[20] Wallach R, Karp RB, Reves JG, Oparil S, Smith LR, James TN. Pathogenesis of paroxysmal hypertension developing during and after coronary bypass surgery: a study of hemodynamic and humoral factors. Am J Cardiol. 1980;46(4):559-565. doi:10.1016/0002-9149(80)90503-2 6998270

[21] Cooper TJ, Clutton-Brock TH, Jones SN, Tinker J, Treasure T. Factors relating to the development of hypertension after cardiopulmonary bypass. Br Heart J. 1985;54(1):91-95. doi:10.1136/hrt.54.1.91 2861835 PMC481855

[22] Li S, Krawczeski CD, Zappitelli M, ; TRIBE-AKI Consortium. Incidence, risk factors, and outcomes of acute kidney injury after pediatric cardiac surgery: a prospective multicenter study. Crit Care Med. 2011;39(6):1493-1499. doi:10.1097/CCM.0b013e31821201d3 21336114 PMC3286600

[23] Ramakrishnan A, Lee LJ, Mitchell LE, Agopian AJ. Maternal hypertension during pregnancy and the risk of congenital heart defects in offspring: a systematic review and meta-analysis. Pediatr Cardiol. 2015;36(7):1442-1451. doi:10.1007/s00246-015-1182-9 25951814 PMC4573362

[24] Ohuchi H, Takasugi H, Ohashi H, . Abnormalities of neurohormonal and cardiac autonomic nervous activities relate poorly to functional status in Fontan patients. Circulation. 2004;110(17):2601-2608. doi:10.1161/01.CIR.0000145545.83564.51 15492308

[25] Tulevski II, Groenink M, van Der Wall EE, . Increased brain and atrial natriuretic peptides in patients with chronic right ventricular pressure overload: correlation between plasma neurohormones and right ventricular dysfunction. Heart. 2001;86(1):27-30. doi:10.1136/heart.86.1.27 11410557 PMC1729810

[26] Alkashkari W, Albugami S, Hijazi ZM. Management of coarctation of the aorta in adult patients: state of the art. Korean Circ J. 2019;49(4):298-313. doi:10.4070/kcj.2018.0433 30895757 PMC6428953

[27] Huynh L, Rodriguez-Lopez S, Benisty K, . Follow-up after neonatal heart disease repair: watch out for chronic kidney disease and hypertension! Pediatr Nephrol. 2020;35(11):2137-2145. doi:10.1007/s00467-020-04621-4 32500246 PMC7515960

[28] Greenberg JH, Parikh CR. Biomarkers for diagnosis and prognosis of AKI in children: one size does not fit all. Clin J Am Soc Nephrol. 2017;12(9):1551-1557. doi:10.2215/CJN.12851216 28667085 PMC5586584

[29] Flynn JT, Kaelber DC, Baker-Smith CM, ; Subcommittee on Screening and Management of High Blood Pressure in Children. Clinical practice guideline for screening and management of high blood pressure in children and adolescents. Pediatrics. 2017;140(3):e20171904. doi:10.1542/peds.2017-1904 28827377

