# Supplementary material for: Long-term Risk of Hypertension After Surgical Repair of Congenital Heart Disease in Children
Source: JAMA Netw Open. 2021 Apr 8;4(4):e215237. doi: 10.1001/jamanetworkopen.2021.5237 (PMC8033439; doi:10.1001/jamanetworkopen.2021.5237)
Supplement: Supplement. — eTable 1. Cohort Build and Baseline Characteristic Codes eTable 2. Outcome Codes eTable 3. ICD-10 CHD Diagnosis and Surgical Billing Codes eTable 4. Health Care Utilization Among Those With Congenital Heart Disease Receiving Surgery vs Matched Controls eTable 5. Long-Term Risk of Hypertension by Type of Congenital Heart Disease eFigure. Patient Selection [file jamanetwopen-e215237-s001.pdf]

Supplementary Online Content

Greenberg JH, McArthur E, Thiessen-Philbrook H, et al. Long-term risk of hypertension after surgical repair of congenital heart disease in children. *JAMA Netw Open*. 2021;4(4):e215237. doi:10.1001/jamanetworkopen.2021.5237

eTable 1. Cohort Build and Baseline Characteristic Codes
eTable 2. Outcome Codes
eTable 3. *ICD-10* CHD Diagnosis and Surgical Billing Codes
eTable 4. Health Care Utilization Among Those With Congenital Heart Disease Receiving Surgery vs Matched Controls
eTable 5. Long-Term Risk of Hypertension by Type of Congenital Heart Disease
eFigure. Patient Selection

This supplementary material has been provided by the authors to give readers additional information about their work.

© 2021 Greenberg JH et al. *JAMA Network Open.*

eTable 1. Cohort Build and Baseline Characteristic Codes

Characteristic/Condition	Database	Codes
**Inclusion Criteria**		
Congenital heart disease	CIHI-DAD<br>OHIP	**ICD-10:** Q250, Q211, Q210, Q218, Q206, Q207, Q208, Q209, Q226, Q228, Q238, Q240, Q241, Q242, Q243, Q244, Q245, Q246, Q255, Q257, Q229, Q239, Q249, Q213, Q201, Q202, Q203, Q205, Q251, Q220, Q221, Q222, Q223, Q212, Q252, Q253, Q254, Q234, Q231, Q260, Q261, Q262, Q263, Q264, Q265, Q266, Q267, Q268, Q269, Q233, Q230, Q200, Q214, Q219, Q224, Q225, Q204, Q232<br>**OHIP:** E650, E660, E660, E670, E671, G268, G269, M108, M132, M134, M137, R703, R705, R709, R712, R713, R715, R716, R717, R718, R720, R721, R722, R723, R724, R725, R726, R728, R729, R735, R736, R737, R738, R742, R743, R746, R748, R749, R750, R751, R753, R761, R754, R756, R757, R758, R759, R762, R768, R770, R771, R772, R774, R781, R790, R799, R801, R808, R814, R815, R818, R820, R826, R827, R830, R841, R857, R863, R870, R874, R876, R920, R921, R922, R923, R925, R926, R927, R928, R929, R930, Z153, Z335, Z341, Z412, Z415, Z428, Z433, Z435, Z443, Z444, Z445
**Exclusion Criteria**		
End-stage renal disease	CIHI-DAD<br>OHIP<br>CORR	OHIP FEECODE: S435, S434, R849, G323, G325, G326, G860, G862, G865, G863, G866, G330, G331, G332, G333, G861, G082, G083, G085, G090, G091, G092, G093, G094, G095, G096, G294, G295, G864, H540, H740
Hypertension	CIHI-DAD<br>OHIP	As defined in outcomes table
**Baseline Characteristics**		
Age	RPDB	N/A
Sex	RPDB	N/A
Year of surgery	OHIP	N/A
Rural residence	RPDB	N/A
Income quintile	RPDB	N/A
Maternal age	MOMBABY<br>RPDB	N/A
Gestational age	MOMBABY	B_GESTWKS_DEL
Birth weight	MOMBABY<br>CIHI-DAD	WEIGHT
Multibirth	MOMBABY	B_MULTIBIRTH
Artificial insemination	OHIP	**OHIP:** G367
Risk adjustment for congenital heart surgery (RACHS) score	OHIP	
Severe congenital heart disease	CIHI-DAD	**ICD-10:** Q212, Q213, Q204, Q201, Q202, Q203, Q200, Q234
Length of stay in hospital	CIHI-DAD	LOS
Length of stay in ICU	OHIP	**OHIP:** C101, G400, G401, G402, G405, G406, G407, G557, G558, G559

© 2021 Greenberg JH et al. *JAMA Network Open.*

Time on ventilation	OHIP	**OHIP:** G557, G558, G559, G405, G406, G407
Dialysis	OHIP	**OHIP:** G082, G083, G085, G090, G091, G092, G093, G094, G095, G096, G295, G294, R849, R850, G323, G325, G326, G860, G862, G863, G865, G866
Acute kidney injury	OHIP	**ICD-10:** N17
Echocardiogram	CIHI-DAD<br>OHIP	**CCI:** 3IP30<br>**OHIP:** G560, G561, G562, G566, G567, G568, G570, G571, G572, G574, G575, G576, G577, G578, G579, G580, G581
Coronary angiogram	CIHI-DAD<br>OHIP	**CCI:** 3IP10, 3IS10<br>**OHIP:** G297, G509
MRI with contrast	OHIP	**OHIP:** X487
Serum creatinine test	OHIP	**OHIP:** L067
Diabetes	CIHI-DAD<br>OHIP	**ICD-10:** E10, E11, E13, E14<br>**OHIP DXCODE:** 250<br>**OHIP FEECODE:** Q040, K029, K030
Pneumonia	CIHI-DAD	**ICD-10:** J12, J13, J14, J15, J16, J17, J18, P23
Turner Syndrome (gonadal dysgenesis)	CIHI-DAD	**ICD-10:** Q96
Down Syndrome	CIHI-DAD	**ICD-10:** Q90
Any chromosomal anomaly	CIHI-DAD	**ICD-10:** Q9
Non-cardiac malformation	CIHI-DAD	**ICD-10:** Q0, Q1, Q3, Q4, Q5, Q6, Q7, Q8
Malformation of the urinary system	CIHI-DAD	**ICD-10:** Q60, Q61, Q62, Q63, Q64

© 2021 Greenberg JH et al. *JAMA Network Open.*

eTable 2. Outcome Codes

Outcome	Dates	Codes Used in Study	Codes Used in Validation	Reference Standard	Operating Characteristics (%)			Reference[a]
					Sensitivity	Specificity	PPV	
Hypertension	1991 to 2002		ICD-9: 401, 402, 403, 404, 405	Algorithm of 2 physician billing claims or 1 hospital discharge with a diagnosis of hypertension in a 2-year period.	72	95	87	Tu et al.[b]
	2002 to 2015	ICD-10: I10, I11, I12, I13, I15	ICD-10: I10, I11, I12, I13, I15					
	2002 to 2015	OHIP DXCODE: 401, 402, 403	OHIP DXCODE: 401, 402, 403, 404, 405					

[a]These validation studies were performed in the adult population. Validations in the pediatric population were not available.
[b] Tu K, Campbell NR, Chen Z, Cauch-Dudek K, McAlister FA. Accuracy of administrative databases in identifying patients with hypertension. Open Medicine 2007 April;1(1):18-26.

© 2021 Greenberg JH et al. *JAMA Network Open.*

eTable 3. *ICD-10* CHD Diagnosis and Surgical Billing Codes

Variable	Code	N=3 600
Post-2002 diagnosis (ICD-10) (first 3 digits presented to allow more broad categorization)	Q21- Congenital malformations of cardiac septa	1 705 (47%)
	Q25- Congenital malformations of great arteries	601 (17%)
	Q20- Congenital malformations of cardiac chambers and connections	585 (16%)
	Q23- Congenital malformations of aortic and mitral valves	194 (5%)
	Q22- Congenital malformations of pulmonary and tricuspid valves	156 (4%)
	Q26- Congenital malformations of great veins	128 (4%)
	Q24- Other congenital malformations of heart	68 (2%)
	P07- Disorders related to short gestation and low birth weight, not elsewhere classified	25 (1%)
	I50- Heart failure	10 (0%)
Post-2002 diagnosis (ICD-10)	Q210- Ventricular septal defect	575 (16%)
	Q213- Tetralogy of Fallot	449 (12%)
	Q212- Atrioventricular septal defect	426 (12%)
	Q251- Coarctation of aorta	377 (10%)
	Q211- Atrial septal defect	237 (7%)
	Q2031- Complete transposition of great vessels	221 (6%)
	Q234- Hypoplastic left heart syndrome	140 (4%)
	Q254- Other congenital malformations of aorta	118 (3%)
	Q201- Double outlet right ventricle	113 (3%)
	Q262- Total anomalous pulmonary venous connection	105 (3%)
	Q2038- Other transposition of great vessels NEC	91 (3%)
	Q244- Congenital subaortic stenosis	57 (2%)
	Q255- Atresia of pulmonary artery	53 (1%)
Surgery billing code (allowing multiple codes during the index hospitalization)	R715- HEART PERI.-CLOSURE-ATRIAL SEPTAL DEFECT	1 191 (33%)
	R718- HEART PERI.-CLOSURE-VENTRICULAR SEPTAL DEFECT	1 109 (31%)

© 2021 Greenberg JH et al. *JAMA Network Open.*

	R754- HEART PERI.-LIGATION OF PATENT DUCTUS-CHILD/INFANT	729 (20%)
	R757- HEART PERI RESECTION COARCTATION-INFANT	429 (12%)
	R759- HEART PERI.-CONGENITAL HEART SHUNT PROC.	374 (10%)
	R725- HEART PERI.-PULMONARY VALVOTOMY & INFUNDIBULAR RESECTION	346 (10%)
	R721- HEART PERI-REPAIR-ARTERIAL-TRANSPOSITION.	326 (9%)
	R720- HEART PERI.-TOTAL REPAIR TETRALOGY OF FALLOT	322 (9%)
	R921- HEART PERI.-REPAIR COMPLETE A/V CANAL	295 (8%)
	R830- ARTERIES-EXC. &/REPAIR-AORTIC ARCH RECONSTRUCTION-INNOMINATE	219 (6%)
	R762- HEART PERI.-CREATION ASD THORACOTOMY	191 (5%)
	R737- HEART PERI.-AORTIC INFUNDIBULAR RESECTION-VENTRICULOMYOTOMY	168 (5%)
	R722- HEART PERI.-REPAIR TOTAL ANOMALOUS PULMONARY VENOUS DRAINAGE	156 (4%)
Surgery billing code (restricting to first code during the index hospitalization)	R715- HEART PERI.-CLOSURE-ATRIAL SEPTAL DEFECT	1 109 (31%)
	R718- HEART PERI.-CLOSURE-VENTRICULAR SEPTAL DEFECT	553 (15%)
	R757- HEART PERI RESECTION COARCTATION-INFANT	312 (9%)
	R720- HEART PERI.-TOTAL REPAIR TETRALOGY OF FALLOT	230 (6%)
	R759- HEART PERI.-CONGENITAL HEART SHUNT PROC.	216 (6%)
	R754- HEART PERI.-LIGATION OF PATENT DUCTUS-CHILD/INFANT	215 (6%)
	R921- HEART PERI.-REPAIR COMPLETE A/V CANAL	162 (4%)
	R721- HEART PERI-REPAIR-ARTERIAL-TRANSPOSITION.	117 (3%)
	R768- HEART PERI.-PULMONARY ARTERY BANDING	95 (3%)

© 2021 Greenberg JH et al. *JAMA Network Open.*

	R722- HEART PERI.-REPAIR TOTAL ANOMALOUS PULMONARY VENOUS DRAINAGE	94 (3%)
	R737- HEART PERI.-AORTIC INFUNDIBULAR RESECTION-VENTRICULOMYOTOMY	85 (2%)
	R717- HEART PERI.-CLOSURE-ANOMALOUS PULMONARY VENOUS DRAINAGE	51 (1%)
	R771- HEART PERI-REPAIR-VASCULAR RING.	45 (1%)
Surgery diagnosis code (restricting to first code during the index hospitalization)	746- Other congenital anomalies of heart	2 930 (81%)
	999- WITHOUT DIAGNOSIS	384 (11%)
	429- All other forms of heart disease	242 (7%)

© 2021 Greenberg JH et al. *JAMA Network Open.*

eTable 4. Health Care Utilization Among Those with Congenital Heart Disease Receiving Surgery vs Matched Controls

Healthcare Utilization Outcome	Status	N	n	%	Total Person-Years of Follow-Up	Incidence Rate Per 10 000 Person-Years	HR (95% CI)			P Value
Pediatrician visit	No CHD	36,000	26,922	74.78%	130,062	2,069.93	1.00 (referent)			<0.0001
	CHD	3,600	3,506	97.39%	225	155,794.53	10.6	10.1	11.1	
Pediatrician/GP visit	No CHD	36,000	35,535	98.71%	10,991	32,330.32	1.00 (referent)			<0.0001
	CHD	3,600	3,514	97.61%	103	340,174.25	4.2	4.1	4.4	
Specialist visit	No CHD	36,000	32,990	91.64%	70,641	4,670.11	1.00 (referent)			<0.0001
	CHD	3,600	3,519	97.75%	89	394,153.23	13.1	12.4	13.8	
Cardiologist visit	No CHD	36,000	3,750	10.42%	339,799	110.36	1.00 (referent)			<0.0001
	CHD	3,600	3,382	93.94%	1,607	21,045.30	131.3	115.6	149.0	
Nephrologist visit	No CHD	36,000	973	2.70%	353,805	27.50	1.00 (referent)			<0.0001
	CHD	3,600	402	11.17%	31,078	129.35	4.7	4.1	5.2	

CHD indicates congenital heart disease, CI, confidence interval, GP, general practitioner, HR, hazard ratio.

© 2021 Greenberg JH et al. *JAMA Network Open.*

eTable 5. Long-Term Risk of Hypertension by Type of Congenital Heart Disease

Outcome	Type of Cardiac Defect	N	No. of events	%	Incidence Rate per 10 000 Person-Years
Hypertension	Ventricular septal defect	574	32	5.6%	58.06
	Tetralogy of Fallot	449	31	6.9%	72.50
	Atrioventricular septal defect	426	28	6.6%	74.51
	Coarctation of aorta	377	72	19.1%	228.79
	Atrial septal defect	234	13	5.6%	66.42
	Complete transposition of great vessels	221	24	10.9%	107.53
	Hypoplastic left heart syndrome	140	49	35.0%	625.57
	Other congenital malformations of aorta	118	15	12.7%	139.07
	Double outlet right ventricle	113	24	21.2%	246.69
	Atresia of pulmonary artery	53	8	15.1%	163.61
Diagnoses not listed above had less than 6 events and cannot be presented in accordance with ICES privacy policy					

© 2021 Greenberg JH et al. *JAMA Network Open.*

eFigure. Patient Selection

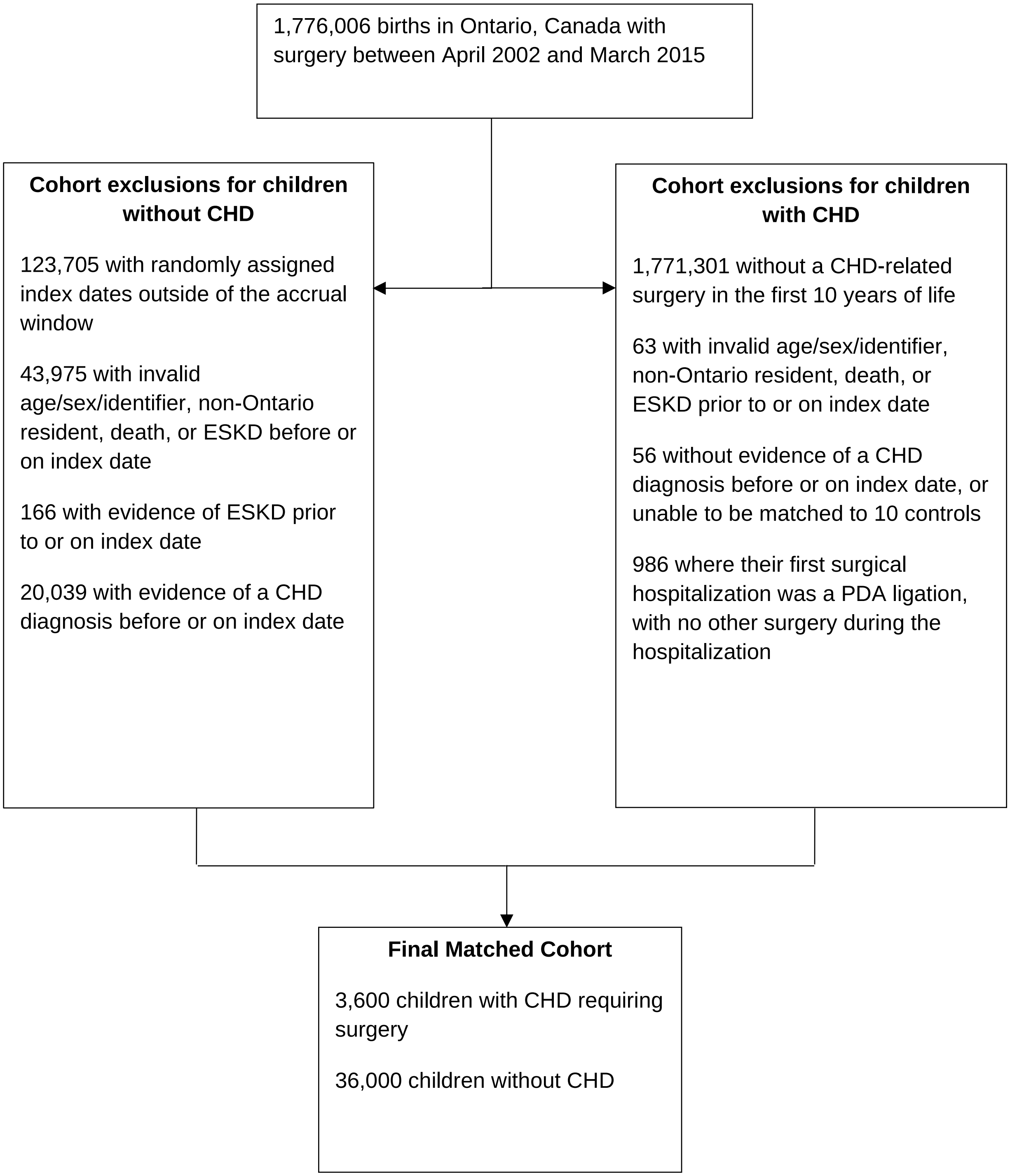

CHD indicates congenital heart disease, ESKD, end-stage kidney disease, PDA, patent ductus arteriosus

© 2021 Greenberg JH et al. *JAMA Network Open.*
